# MultiMSOAR 2.0: An Accurate Tool to Identify Ortholog Groups among Multiple Genomes

**DOI:** 10.1371/journal.pone.0020892

**Published:** 2011-06-21

**Authors:** Guanqun Shi, Meng-Chih Peng, Tao Jiang

**Affiliations:** Department of Computer Science, University of California Riverside, Riverside, California, United States of America; University of Leuven, Belgium

## Abstract

The identification of orthologous genes shared by multiple genomes plays an important role in evolutionary studies and gene functional analyses. Based on a recently developed accurate tool, called MSOAR 2.0, for ortholog assignment between a pair of closely related genomes based on genome rearrangement, we present a new system MultiMSOAR 2.0, to identify ortholog groups among multiple genomes in this paper. In the system, we construct gene families for all the genomes using sequence similarity search and clustering, run MSOAR 2.0 for all pairs of genomes to obtain the pairwise orthology relationship, and partition each gene family into a set of disjoint sets of orthologous genes (called *super ortholog groups* or *SOGs*) such that each SOG contains at most one gene from each genome. For each such SOG, we label the leaves of the species tree using 1 or 0 to indicate if the SOG contains a gene from the corresponding species or not. The resulting tree is called a *tree of ortholog groups* (or *TOGs*). We then label the internal nodes of each TOG based on the parsimony principle and some biological constraints. Ortholog groups are finally identified from each fully labeled TOG. In comparison with a popular tool MultiParanoid on simulated data, MultiMSOAR 2.0 shows significantly higher prediction accuracy. It also outperforms MultiParanoid, the Roundup multi-ortholog repository and the Ensembl ortholog database in real data experiments using gene symbols as a validation tool. In addition to ortholog group identification, MultiMSOAR 2.0 also provides information about gene births, duplications and losses in evolution, which may be of independent biological interest. Our experiments on simulated data demonstrate that MultiMSOAR 2.0 is able to infer these evolutionary events much more accurately than a well-known software tool Notung. The software MultiMSOAR 2.0 is available to the public for free.

## Introduction

The ever-increasing number of completely sequenced genomes brings great opportunities as well as challenges to the study of comparative genomics. It makes the study of the evolutionary history of closely related species at the genome level possible. It also enhances our ability to perform gene functional analyses across different species. For these purposes as well as many other applications, the identification of orthologous genes across different species often serves as a starting point.

### Definitions

Orthologous genes (*i.e., orthologs*) are genes in different genomes that evolved from a common ancestral gene through speciation events [1]. They are more likely to preserve the original gene function. As a result, orthologs are often used as universal and unique landmarks within each genome as well as links across different genomes [2].

Orthology between two genomes is usually thought of as a many-to-many relationship due to post-speciation gene duplications [3]. However, if we know which genes are the direct descendants of the ancestral genes and which are duplicated after the speciation, then we can define a one-to-one orthology relationship between the two direct descendant genes of each ancestral gene (such a pair of genes are said to form an *ortholog pair*), while treating the duplicated genes as inparalogs [4], [5].

**Figure 1:**
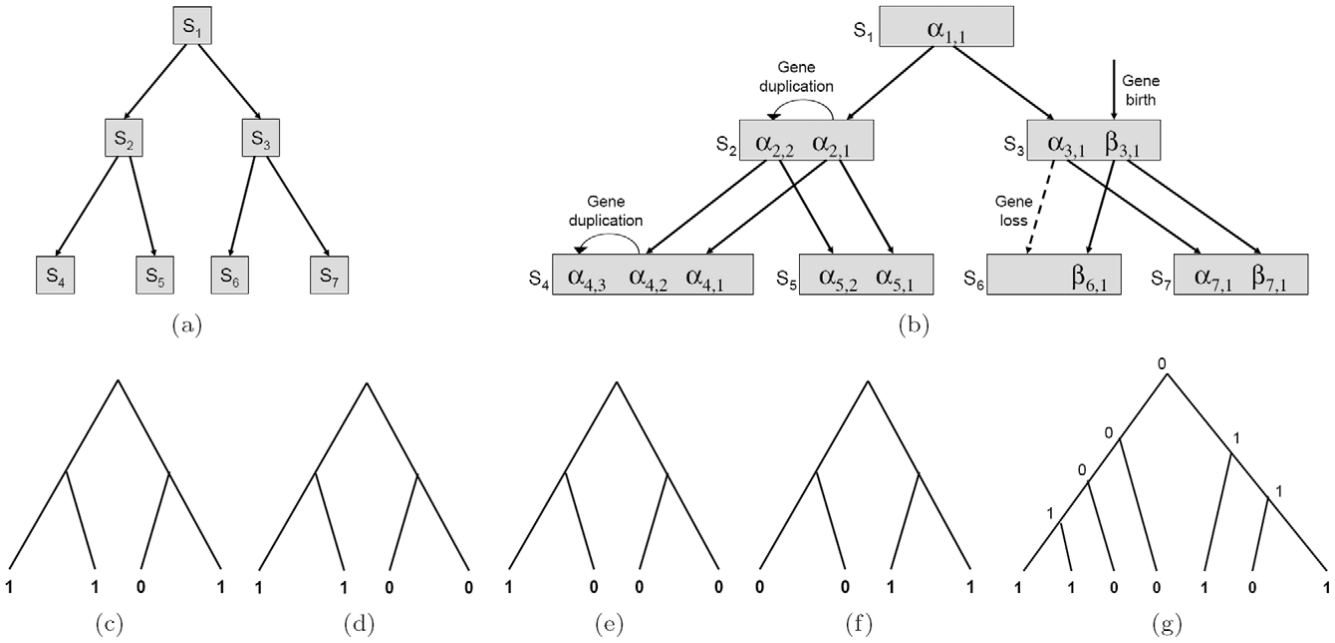
An example of genome evolution and TOGs. (a) The species tree for four species: 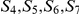. (b) An example of genome evolution for the four species in (a). (c) The TOG for genes 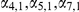 in (b). (d) The TOG for genes 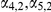 in (b). (e) The TOG for gene 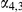 in (b). Note that, in this paper, we will only be interested in ortholog groups containing at least two genes, and singleton ortholog groups will be ignored since they consist of only inparalogs from individual genomes. (f) The TOG for genes 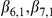 in (b). (g) An example of a TOG labeling. The labeling suggests two ortholog groups in the TOG, one consisting of two genes from the two leftmost species and the other two genes from the last three species.

When multiple genomes are being compared, the orthology relationship is more complicated because of the interleaving between speciation and gene duplication events. In this paper, we extend the above one-to-one orthology relationship between a pair of genomes to multiple genomes in a straightforward way and define an *ortholog group* for a given set of genomes as a maximal set of genes (from different genomes) that are the direct descendants of the same ancestral gene. Note that the genes in such an ortholog group are not separated by any gene duplication. Hence, this definition, although a bit stringent, is faithful to the original definition of orthology in Ref. [1]. For example, according to this definition, there are 4 ortholog groups in Figure 1(b): (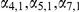), (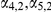), (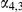), (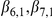). We note in passing that other more general definitions of ortholog groups have been considered in the literature and used in popular orthology databases such as COG [6] and EnsemblCompara [3]. In these definitions, orthology is considered as a many-to-many relationship and thus paralogs (*i.e.*, genes that are separated by duplications) are often allowed in an ortholog group. We prefer treating orthology as a one-to-one relationship because it makes the presentation of the paper simpler and validation of our results cleaner. Moreover, the one-to-one orthology relationship can be thought of as a refinement of the more general many-to-many relationship.

### Existing Ortholog Assignment Tools

Most of the traditional ortholog identification methods are based on sequence similarity search, such as COG/KOG [6], OrthoMCL [7], InParanoid/MultiParanoid [4], [8] and HomoloGene [9]. Generally speaking, these methods first calculate some pairwise similarity scores and then use some clustering algorithms to identify ortholog pairs or groups. Take the InParanoid program for example. It assigns a gene pair with the bidirectional best hit (*i.e., BBH*) as a main ortholog pair and uses it as the “seed” to cluster similar genes from both genomes into an ortholog group. As its extension to multiple genomes, the MultiParanoid program basically clusters the pairwise orthology results of InParanoid to generate ortholog groups for multiple genomes. Though the BBH requirement for a main ortholog pair seems to be reasonable when comparing two genomes, it becomes too stringent when comparing multiple genomes. As a result, the MultiParanoid program may miss a lot of true ortholog groups when some of the ortholog pairs are not BBHs. OrthoMCL is an ortholog assignment program similar to InParanoid, but uses a different clustering algorithm (the Markov Clustering algorithm, or *MCL*) to find ortholog groups for multiple genomes. However, it cannot resolve the many-to-many orthology relationship among multiple genomes effectively. As a result, the ortholog groups found by OrthoMCL may include lots of “recent” inparalogs from each genome [7].

Another popular method to identify orthologs is based on phylogenetic trees, such as TreeFam [10], PhyOP [11], and EnsemblCompara GeneTrees [3]. A phylogeny can be used conveniently to represent the evolution of a gene family. However, tree-based methods generally present orthology as a many-to-many relationship. Most of them can never tell the “parent-daughter” relationships among duplicated genes [12]. As a result, most tree-based methods cannot differentiate orthologs that are direct descendants of an ancestral gene and those inparalogs that are products of recent duplications. Consequently, each ortholog group found by these methods tends to include lots of lineage-specific duplicated inparalogs.

By taking other information into consideration, such as gene positions and genome rearrangement, some combinatorial approaches have been proposed in recent years. CCCPart is a synteny-based approach to find orthologs based on the assumption that isofunctional genes are well preserved both in common gene neighborhood as well as in sequence similarity between two or more species [13], [14]. However, it is known that genome rearrangement is very common between closely related genomes [15]–[18]. In fact, there might be many microrearrangments even within the same synteny block [17]. Based on genome rearrangement, a high-throughput ortholog assignment system called MSOAR [19] has been developed. It is based on the assumption that orthologs should correspond to each other on the evolutionary path that minimizes the number of rearrangements and post-speciation duplications. By dealing with tandem gene duplications explicitly using a phylogenetic approach, an improved system MSOAR 2.0 was recently reported in Ref. [5], which has been shown to outperform the original system MSOAR in terms of prediction accuracy. However, MSOAR and MSOAR 2.0 can only assign orthologs between two genomes. As an extension to MSOAR, MultiMSOAR tries to assign orthologs among multiple genomes by using a simple clustering method based on the pairwise results of MSOAR [20]. However, the MultiMSOAR program can actually handle only three genomes well. When more genomes are involved, MultiMSOAR may not find ortholog groups accurately because it does not take into account the phylogenetic relationship among the genomes. Furthermore, MultiMSOAR only considers those ortholog clusters that do not have gene losses in any species to be ortholog groups. This constraint might be acceptable for three closely related species, but it is too stringent when considering more species, since we expect to see many gene births and losses as well as duplications in the evolutionary history. As a consequence, we should allow gene losses within an ortholog group and ortholog groups to be composed of genes from a subset of the genomes.

### Current Work

In this paper, we develop a system called MultiMSOAR 2.0 to identify ortholog groups for multiple genomes. In addition to being an extension of MSOAR 2.0 to multiple genomes, MultiMSOAR 2.0 presents a new combinatorial approach for constructing ortholog groups. Compared with MultiMSOAR, MultiMSOAR 2.0 allows gene losses within an ortholog group and ortholog groups involving genes only from a subset of the genomes. It also attempts to minimize the number of gene births, losses and duplications within a gene family when assigning ortholog groups. Moreover, compared with many other ortholog assignment tools for multiple genomes, MultiMSOAR 2.0 can provide more information about genome evolution in terms of gene births, losses as well as duplications.

**Figure 2:**
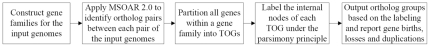
An outline of MultiMSOAR 2.0.

An outline of MultiMSOAR 2.0 is shown in Figure 2. In short, MultiMSOAR 2.0 constructs gene families for all the genomes first by using sequence similarity search (*i.e.*, BLASTp) and the clustering algorithm MCL as done in Ref. [5]. Then it applies MSOAR 2.0 to find ortholog pairs between all pairs of genomes. After that, it builds a weighted multipartite graph using the pairwise orthology information and sequence similarity between each pair of orthologs and attempts to find a maximum weight matching for each gene family. Then it partitions each family into a set of disjoint sets of orthologous genes (called *super ortholog groups* or *SOGs*) such that each SOG contains at most one gene from each genome. Each such SOG may potentially consist of several ortholog groups. In order to partition a SOG into ortholog groups, MultiMSOAR 2.0 labels the leaves of the species tree using 1 or 0 to indicate if the SOG contains a gene from the corresponding species or not. The resulting tree is called a *tree of ortholog groups* (or *TOGs*). MultiMSOAR 2.0 then employs one of the two algorithms devised in this paper (called the *NodeCentric* and *TreeCentric* algorithms) to label the internal nodes of each TOG based on the parsimony principle and some biological constraints. Ortholog groups can then be trivially identified from each fully labeled TOG. The details of each of the main steps in Figure 2 are explained in the Methods section. Note that each ortholog group found by MultiMSOAR 2.0 is contained in some TOG but a TOG may contain several ortholog groups. An example is shown in Figure 1(g), where the TOG contains two ortholog groups and the second ortholog group contains a gene loss.

## Methods

### Homology Search and Gene Family Construction

Since we have multiple genomes, we define a gene family to consist of all homologous genes on all the genomes under study. As in Ref. [5], [19], only protein coding genes will be considered. For genes with alternative splicing variants, we use their longest transcripts. Similar methods have been used in previous studies [5], [21]. To cluster all the genes into gene families, we combine all protein sequences from all genomes together, and perform an all-vs-all BLASTp homology search [22]. Then we use the popular clustering program MCL [23] to construct gene families. Similar methods have been used in many other papers [7], [10], [21].

### Pairwise Genome Comparison

Since we try to identify ortholog groups among multiple genomes based on pairwise comparison, the prediction accuracy of ortholog pairs between two genomes is critical for the performance of our multiple genome system. MSOAR 2.0 has shown to be the most accurate prediction tool for assigning one-to-one ortholog pairs between two closely related genomes [5]. So, it is preferable to use the output of MSOAR 2.0 as the input to our current system. For a comparison among 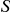 genomes, we apply MSOAR 2.0 to all pairs of the 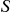 genomes, and use the 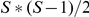 pairwise comparison results to define a multipartite for each gene family to be partitioned in MultiMSOAR 2.0.

### Partition of Each Gene Family into TOGs

In our definition of ortholog groups, each group may include at most one gene from each genome. However, a gene family may include many homologous genes from each genome (*i.e.*, paralogs), making it necessary to split the genes in a family into TOGs, such that each TOG contains at most one gene from every genome. This is done by employing a heuristic maximum weight 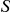-dimensional matching algorithm as follows. Similar methods have been used in Ref. [20], [24].

Suppose we have 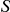 genomes, 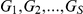, where 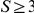. For a given gene family, the number of genes from each genome are denoted as 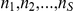. We can construct an 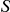-partite (or 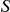-stage) graph 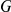 with 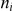 (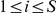) vertices in the part corresponding to genome 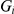 (called stage 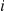). We add edges to 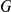 by using the pairwise orthology information produced by MSOAR 2.0. Specifically, we add an edge between two vertices in 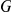 if and only if the corresponding two genes are from two different genomes and they are assigned as an ortholog pair by MSOAR 2.0. We assign a weight to such an edge, which is the BLASTp similarity score between the ortholog pair.

Since we would like to obtain a perfect 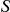-dimensional matching with the maximum weight among the 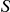 stages, we need to add some dummy vertices to some of the stages in 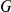 to make them all have the same number of vertices. Let 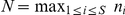 be the maximum number of paralogs on any genome in the gene family. Then we add 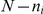 (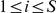) dummy vertices to the 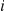-th stage. The maximum (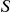-dimensional) matching problem for 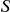-partite graphs (where 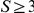) is known to be NP-hard [25], and 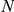 could be large for a real gene family when a large number of genomes are considered. So, we will use a heuristic optimization approach to find a good matching. Since the maximum weight matching for a bipartite graph can be computed by the Hungarian algorithm in cubic time [26], we first find a maximum weight bipartite matching for two stages in 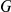, combine them into one stage, and apply the Hungarian algorithm iteratively on the remaining stages in 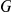 until only one stage is left. This results in a matching for the original 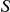-partite graph 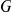. This approach is very similar to the method used in MultiMSOAR [20], except that we use a post-order traversal on the species tree to decide the order that stages are combined. This way, a stage is always combined with another stage that is close to it on the species tree. Another difference is that we use the bit score as the weight of an edge in 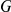. If there is no edge between two vertices in different stages, we deem that there is an edge with weight 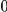 between them.

An example of the gene family partition is shown in Figure 1, where the figures in (c), (d), (e) represent 3 TOGs for the 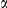 gene family while Figure 1(f) represents a single TOG for the 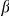 gene family.

### Labeling of TOGs

In order to identify ortholog groups within a TOG, we need to label the internal nodes (which correspond to ancestral genomes) using binary representations as well. Here, 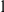 means that the a gene is present in the corresponding ancestral genome while 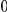 means absence. Two constraints will be assumed:

1. *Intratree constraint*: If node 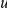 is labeled with a 0 and 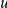 has an ancestral node that is labeled with a 1, then every descendant node of 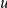 must be labeled with a 0.
2. *Intertree constraint*: Suppose that 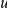 and 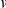 are two nodes such that each of them is labeled with a 1 in at least one TOG. Then every node on the path connecting 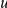 and 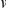 must be labeled with a 1 in at least one TOG.

The intertree constraint makes sure that no gene is born twice in evolution, which is a commonly accepted hypothesis in molecular evolution since double gene birth events are extremely rare. The intratree constraint follows from the definition of orthology (that orthologs evolved through speciation only).

Among all the labelings of the TOGs satisfying the above two constraints, we would like to find one that minimizes the number of gene births, duplications and losses in the evolution of the family. Since each edge of a TOG whose nodes are labeled with 01 or 10 represents a gene birth/duplication or a gene loss, we need to find a parsimonious way to label the internal nodes so that the number of 01 or 10 edges is minimized. For simplicity, let us call a 01 or 10 change on an edge a *flip*.

We can now formulate the TOG labeling problem as a combinatorial optimization problem as follows:

***TOG Labeling:*** *Given* 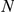 *TOGs, find a binary labeling of all the internal nodes of the TOGs so that both intratree and intertree constraints are satisfied and the total number of flips is minimized.*

The problem can be solved by a trivial exhaustive search algorithm that considers all possible labelings of the TOGs. However, since a binary tree with 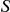 leaves has 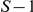 internal nodes, this algorithm runs in time O(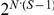), which is impractical even if 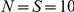. We need to find more efficient solutions to this problem.

Before we proceed with our algorithms, we first prove the following two lemmas, which will help accelerate the speed of our labeling algorithm.

**Lemma 1** *If two child nodes are labeled as* 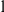 *, then in any optimal labeling, their parent node must be labeled as* 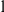 *.*

*Proof*. Suppose that in an optimal labeling 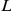, an internal node 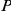 is labeled as 0 in some TOG but both of its children are labeled as 1. If we change the label of 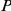 to 1, the two constraints will not be violated, and there will be two fewer flips on the two edges from 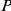 to its two children. Even if this change might incur a new flip on the edge from 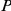 to its parent node, the total number of flips will still be reduced. This is a contradiction to the assumption that 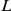 is an optimal labeling, which completes our proof.

**Lemma 2** *If two child nodes are labeled as* 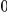 *, then there is an optimal labeling, where their parent node is labeled as* 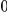 *.*

*Proof*. Suppose that an internal node 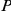 of some TOG 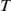 is labeled as 1 while both of its children are labeled as 0 in some optimal labeling. If we change the label of 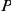 to 0, it is easy to see that the intratree constraint will not be violated. However, the intertree constraint might be violated if the node 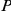 is also labeled as 0 in all other TOGs. Then, according to Lemma 1, the two child nodes of 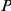 cannot be labeled as 1 at the same time in each of the other TOGs. If each of the two child nodes of 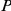 is labeled as 0 in all other TOGs, then we are safe to change the label of 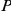 from 1 to 0 in the TOG 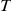 since the change will not violate the intertree constraint. Otherwise, there is at least one TOG 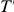, in which the two child nodes of 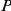 are labeled as 0 and 1, respectively. In this case, we can change the label of 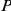 in 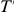 to 1. From the proof of Lemma 1, we know that changing the label of 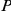 in 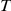 will decrease the number of flips by at least 1, while changing the label of 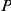 in 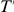 may increase the number of flips by at most 1. If we change the labels of node 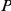 is TOGs 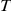 and 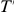 simultaneously, the total number of flips will not increase and thus the labeling is still optimal. Moreover, such a simultaneous change will keep the intertree constraint satisfied. This completes the proof of Lemma 2.

The TOG labeling problem is trivial to compute without the intratree and intertree constraints. If we only consider the intratree constraint, the problem can still be solved by using dynamic programming in polynomial time. However, the intertree constraint makes the problem much harder. Here, we propose two different algorithms to solve the TOG labeling problem: the *NodeCentric* algorithm and the *TreeCentric* algorithm. The algorithms are sketched below.

The basic idea behind the NodeCentric algorithm is to label all 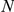 TOGs simultaneously by dynamic programming. In other words, it labels each internal node of the species tree with a binary vector of 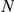 bits. In order to keep track of the validity of the two constraints, we will use label 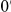 (when considering some TOG) to indicate that (i) the current node is labeled as 0 in the TOG and (ii) some descendant of the current node is labeled as 1 in the TOG. Thus, the label 0 now means that all descendant nodes are also labeled as 0. The algorithm proceeds in post-order. For each internal node 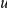 in the species tree, it enumerates all possible label vectors at 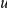 and for each vector, it computes the minimum number of flips in the subtree under node 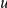 by considering all feasible label vectors of its two children without violating the two constraints. By Lemmas 1 and 2, we can quickly fix the label of 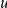 in a TOG if the labels of its two children in the same TOG are both fixed as 0 or both fixed as 1.

Since the left and right children can be considered separately, it seems that the above algorithm would run in 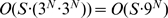 time, which could be impractical if 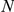 is large. However, with a careful analysis, we find that at most 3 (instead of 9) combinations of the parent-child labels are possible in a TOG. If the parent label is fixed as 0, then the child label must be fixed as 0 as well. Otherwise, the parent label could be 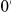 or 1. If it is 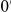, then the child label could be either fixed as 0 or one of 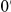 and 1. If the parent label is 1, then the child label must be fixed either as 0 or as 1 due to the intratree constraint. So, in any case, at most 3 combinations of the parent-child labels should be considered in a TOG and hence, a total number of 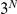 values need to be computed. The intertree constraint may reduce the number of legal combinations even further. This implies an efficient implementation of the NodeCentric algorithm with time complexity 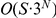.

While the NodeCentric algorithm goes through each node sequentially, the TreeCentric algorithm goes through each TOG sequentially. For a subset of fully labeled TOGs on the same species tree, the *union TOG* is a fully labeled TOG obtained by taking the Boolean *or* operation on the labels of each given TOG at the same node of the species tree. Let us order the TOGs arbitrarily as 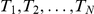. For each TOG 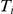, the TreeCentric algorithm enumerates all feasible binary labelings of the TOG 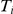 by taking into account the intratree constraint. This can be done efficiently by dynamic programming. For each such labeling of 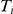, it enumerates all possible union TOGs 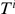 covering 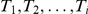, and then computes and records the minimum number of flips in the TOGs 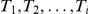 for each union TOG 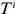, by taking advantage of the previously recorded minimum number of flips in 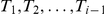 for each union TOG 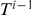. Finally, the minimum number of flips in all TOGs 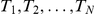 is obtained by considering all possible union TOGs covering 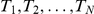 and taking into account the intertree constraint. Since the number of different union TOGs is 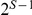, the above algorithm runs in 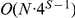 time.

More detailed pseudocodes of both algorithms are given in Algorithms 1 and 2. For the convenience of the reader, we list the notations used in the algorithms and their brief explanations explicitly below.

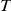
: the species tree.

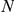
: the number of TOGs in a gene family.

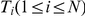
: the TOGs in the gene family.

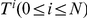
: the union TOG covering TOGs 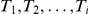.

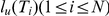
: the label of node 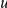 in 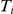.

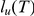
: the label vector of node 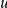 in 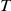 with 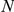 bits, where the 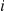-th bit is 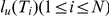.

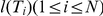
: the labeling of TOG 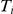.

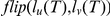
: the number of flips (*i.e.*, Hamming distance) between two labelings 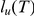 and 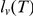.

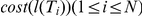
: the number of flips in 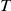 when labeled as 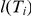.

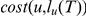
: the total number of flips in the subtree of 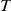 rooted at 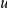 with labeling 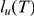.

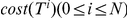
: the total number of flips in the first 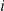 TOGs when their labelings satisfy the intratree constraint and form the union TOG 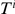.

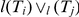
: the boolean *or* operation between labelings 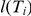 and 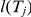.

**Algorithm 1** NodeCentric (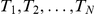)

1: Traverse 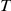 in post-order

2: **for all** node 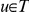 do

3: **if** 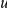 is a leaf node **then**

4:  
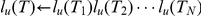


5:  
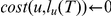


6: **else**

7:  **for all** possible labeling 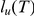 at node 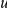 **do**

8:   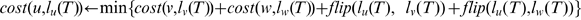, where 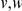 are the two child nodes of 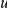, and 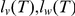 are their labelings such that 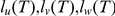 satisfy the two constraints

9:  **end for**

10: **end if**

11: **end for**

12: Traverse *T* in pre-order and retrieve the labeling of each node that gave rise to the minimum cost by a standard backtracing

Both algorithms NodeCentric and TreeCentric are exponential time algorithms. However, in practice, the number of genomes in comparison is expected to be small (usually 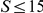). So we can use the TreeCentric algorithm to find an optimal TOG labeling efficiently. When the value of 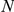 is smaller, it is faster to apply the NodeCentric algorithm. Note that, the two algorithms may find different labelings for the same input, both of which are optimal.

**Algorithm 2** TreeCentric 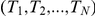


1: Initialize union TOG 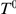 by labeling 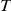 with 0's

2: 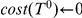


3: **for** 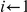 to 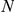 do

4: **for all** union TOG 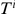 **do**

5:  
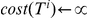


6: **end for**

7: **for all** labeling 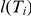 **do**

8:  **if** 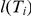 satisfies the intratree constraint then

9:   **for all** union TOG 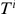 **do**

10:    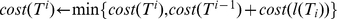, where 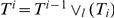


11:   **end for**

12:  **end if**

13: **end for**

14: **end for**

15: Let 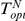 denote a union TOG that minimizes 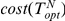 and satisfies the intertree constraint Traverse the TOGs in reverse order ( *i.e.*, 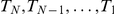) and retrieve the optimal labeling for each TOG 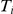 that gave rise to 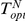 by a standard backtracing.

### Ortholog Group Identification

After labeling all TOGs, it is straightforward to identify ortholog groups. Starting from the root of each TOG, we can find the highest ancestral nodes labeled as 1. All genes at the descendent leaves of such an ancestral node form an ortholog group. An example is shown in Figure 1(g). In addition, with the labeling of each TOG, we can easily identify evolutionary events including gene births and losses as well as duplications. For each edge in the TOG, if the parent-child labeling is 1-0, then there is a gene loss. If the labeling is 0-1, and the parent node is labeled as 0 in all other TOGs, then it represents a gene birth. Otherwise, it represents a gene duplication.

## Results

In order to test the performance of our system MultiMSOAR 2.0, we first apply it to simulated data, and compare it with the popular ortholog assignment tool MultiParanoid [27] for multiple genomes. For real data experiments, besides comparison with MultiParanoid, we also compare our results with Roundup [28], which is a well known multi-genome repository of orthology information and the Ensembl ortholog database.

### Simulation Results

Our simulation test is an extension of the one in Ref. [5] for testing the performance of MSOAR 2.0. However, we now need to simulate more genome evolutionary events, including gene mutations, gene births, gene duplications, gene losses, genome rearrangements (including reversals, translocations, fusions and fissions) and speciations (graphical examples of these events are shown in Figure S2 in Materials S1). To make things easier, we only simulate the evolution of 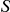 (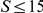) single-chromosomal genomes as done in Ref. [5]. In order to generate 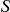 contemporary genomes, we first generate a random species tree 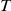 with 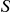 leaf nodes. Each internal node in 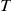 represents an ancestral genome while the leaf nodes represent the current genomes. Each edge in 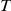 represents a speciation event. We then randomly generate a genome with 100 genes consisting of 3,000 nucleotides each at the root of 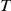. For each speciation event, we simulate 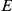 evolutionary events, which include 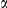 gene duplications, 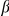 gene births, 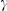 gene losses, and 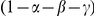 genome rearrangements. To generate the gene duplications, we randomly choose a gene, copy it and insert it into the genome next to the original copy or at a random position, depending on whether the duplication is tandem or random (here we assume 50% of all duplications are tandem, as done in Ref. [5]). To simulate the birth of a new gene, we create a new gene and randomly insert it into the genome. To simulate the loss of a gene, we randomly choose a gene and delete it from the genome. For genome rearrangements, since there is only one chromosome, only reversals are considered. Reversals are simulated by randomly choosing two positions on the genome and reverse all the genes between them.

To simulate gene (point) mutations, we use a popular sequence simulation tool *evolver* from the PAML package [29]. By running *evolver* with default options on the codon sequence at the root of a branch, we can obtain the mutated codon sequence over a pre-specified branch length 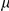. Since branch length can be measured in terms of the expected number of substitutions per site, we may use 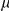 to control the mutation rate of a gene. We assume that between every two (genome-level) evolutionary events, all the genes on the existing genomes evolve at the same rate. In other words, a molecular clock is assumed.

In summary, our simulation data is controlled by a 6-parameter set: 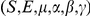, where 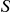 is the number of species, 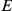 the total number of evolutionary events after each speciation, 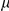 the gene mutation rate, and 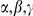 the percentages of gene duplications, births and losses among the 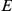 events, respectively.

**Figure 3:**
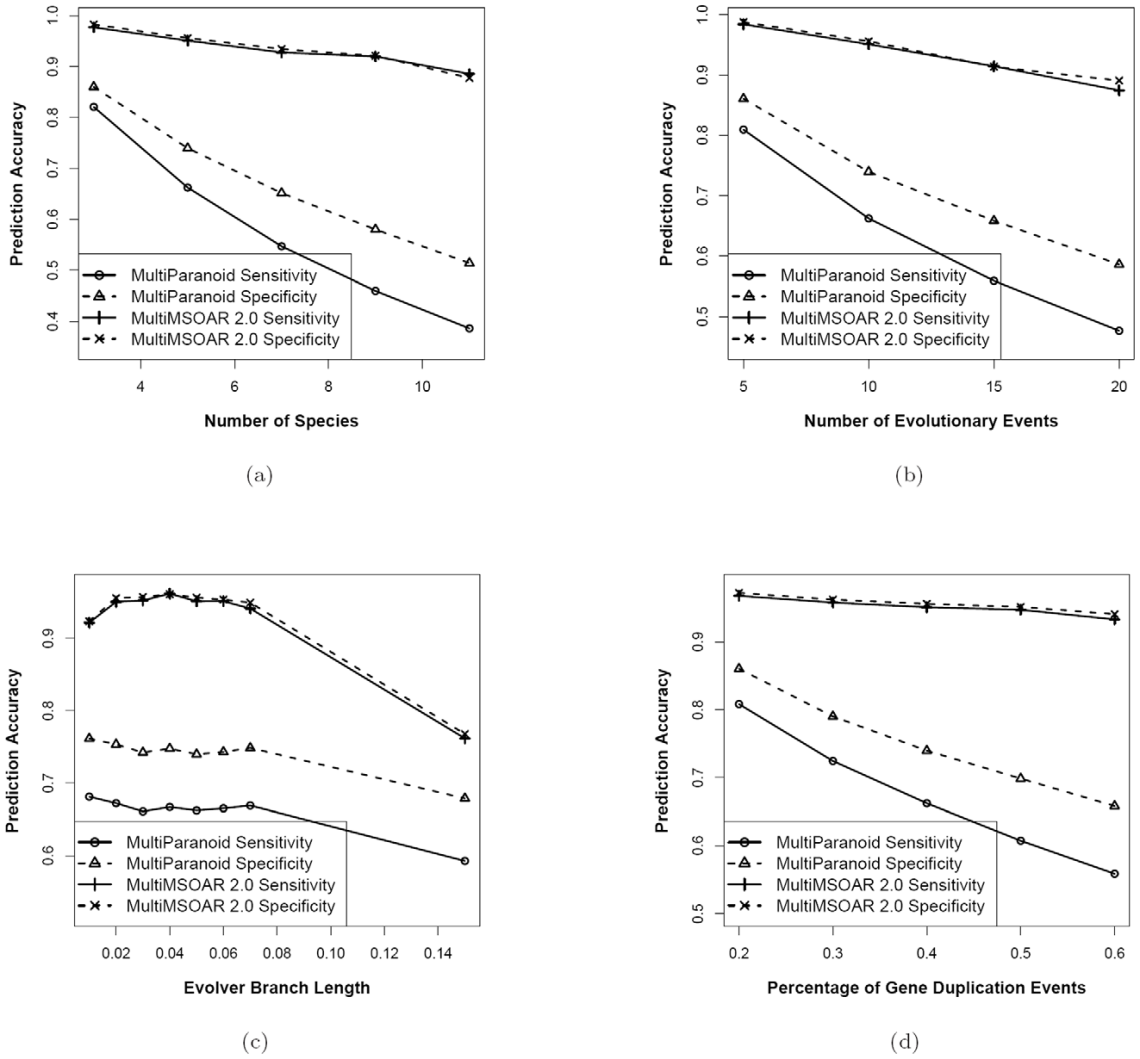
Comparison of MultiMSOAR 2.0 and MultiParanoid on simulated data. (a) Simulation results on the parameter set 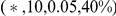 where the parameter 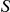 is varied. (b) Simulation results on the parameter set 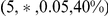 where the parameter 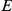 is varied. (c) Simulation results on the parameter set 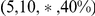 where the parameter 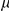 is varied. (d) Simulation results on the parameter set 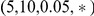 where the parameter 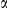 is varied.

To study the effects of different parameters on the performance of MultiMSOAR 2.0, we set the default values for each parameter as 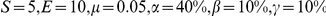, and we will vary one parameter at a time. To measure the prediction accuracy, we use two popular measurements: *sensitivity* and *specificity*. Here, sensitivity is defined as the number of the true ortholog groups (*i.e.*, true positives) identified by a program divided by the total number of known ortholog groups, and specificity is defined as the number of true ortholog groups identified divided by the number of ortholog groups output. We compare the ortholog groups found by MultiMSOAR 2.0 and MultiParanoid. In order for an identified ortholog group to be a true positive (*i.e., TP*), we require that all genes in the identified ortholog group match exactly with all the genes in a known ortholog group. For each parameter set, we generate 10 simulated data sets and run MultiMSOAR 2.0 and MultiParanoid on these data respectively. Finally we calculate the average prediction accuracies of the two programs on each parameter set. The prediction accuracies of the two programs are shown in Figure 3.

Figures 3(a), (b), (d) show that with the increase of the number of species, the number of evolutionary events, and the number of gene duplications, the prediction accuracies of both programs decrease since it becomes harder for them to correctly identify ortholog groups. However, we notice that the decrease in accuracy for MultiMSOAR 2.0 is mild while the decrease is sharp for MultiParanoid, especially in Figure 3(d). This could be because when more genes are duplicated, it becomes increasingly difficult for MultiParanoid to decide if a duplication happened in an ancient genome or in a more recent genome. Thus, it might confuse some ancient duplications with recent duplications and miss calling some true ortholog groups. On the other hand, MultiMSOAR 2.0 infers the time of each duplication explicitly when labeling TOGs, and is thus more resilient to the increase of gene duplication events. However, since the labeling algorithm used in MultiMSOAR 2.0 is based on the parsimony principle and the optimal labeling might not be unique, the actual labeling given by MultiMSOAR 2.0 may not necessarily reflect the true evolutionary history. As a result, when the number of gene duplications increases, the prediction accuracy of MultiMSOAR 2.0 also decreases, but much more slowly than in the case of MultiParanoid.

Figure 3(c) is interesting and deserves some explanation. With the increase of the branch length 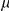 defined in *evolver* from 0.01 to 0.04, both the sensitivity and specificity of MultiMSOAR 2.0 increase a little bit. This is because when 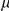 increases, it becomes slightly easier for MultiMSOAR 2.0 to differentiate duplicated genes from their original copies based on sequence similarity. However, when 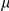 goes from 0.07 to 0.15, the prediction accuracies of both programs sharply decrease. This is because the sequence similarity between homologous genes originated from a common ancestral gene becomes weaker with the increase of 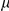. As a result, it becomes harder for MultiParanoid to identify ortholog groups solely based on sequence similarity, and for the MCL algorithm used in MultiMSOAR 2.0 to correctly cluster homologous genes into a gene family. Without correct gene families, we cannot expect MultiMSOAR 2.0 to find the ortholog groups correctly.

Generally speaking, from the four figures above, we can see that the prediction accuracy of MultiMSOAR 2.0 is significantly higher than that of MultiParanoid. With more species, more evolutionary events and more gene duplications, the advantage of MultiMSOAR 2.0 over MultiParanoid becomes more apparent. Besides, in the simulation, MultiMSOAR 2.0 is always able to achieve more than 90% prediction accuracy (in terms of sensitivity and specificity) as long as the gene mutation rate is not too high. This is pretty remarkable considering the large number of species and evolutionary events involved. Moreover, MultiMSOAR 2.0 can provide more information about gene births, losses and duplications in addition to identifying ortholog groups. In the simulation experiments, we also tested the accuracy of MulitMSOAR 2.0 in inferring gene births, losses and duplications, and compared its performance with Notung, a well-known software tool for reconciling genes trees with species trees by taking into account gene duplication and loss events [30], [31]. Since Notung does not consider gene births, we only compare the sensitivity and specificity of MultiMSOAR 2.0 and Notung with respect to gene duplication and loss events. It turns out that the prediction accuracies of MultiMSOAR 2.0 on duplications and losses are generally much higher than those of Notung. Due to the page limit, the prediction accuracies concerning these events by MultiMSOAR 2.0 and Notung on simulated data are summarized in Tables S1, S2, S3, S4 in Materials S1. Note that Notung fails to detect most gene losses because it prunes the species tree when an entire gene family is missing in a genome.

### Real Data Experiments

Since MultiMSOAR 2.0 is a tool to identify ortholog groups for multiple genomes that are closely related on a genome scale, to test its performance on real data, we choose to use the mammalian genomes that have been completely sequenced. We downloaded seven mammalian genomes from the Ensembl genome browser (http://www.ensembl.org/): human (*Homo sapiens*), chimpanzee (*Pan troglodytes*), macaque (*Macaca mulatta*), mouse (*Mus musculus*), rat (*Rattus norvegicus*), cow (*Bos taurus*) and opossum (*Monodelphis domestica*) (version 57, March 2010). The species tree for the seven mammalian genomes is downloaded from Ensembl as well.

For the purpose of comparison, we choose to compare the results of MultiMSOAR 2.0 with those of the popular tool MultiParanoid, Roundup and the Ensembl ortholog database. For MultiParanoid, we deem all the genes in the same cluster output by the program as an ortholog group assigned by MultiParanoid. We run MultiMSOAR 2.0 and MultiParanoid on the real data sets respectively and compare their results. Roundup is a recently developed multi-genome repository of orthologs for over 250 genomes [28]. We download the ortholog groups for the concerned genomes from its website (http://roundup.hms.harvard.edu/). Since Roundup uses genomes from different sources, we need to map the genes used in Roundup to the corresponding genes used in Ensembl. For the Ensembl ortholog database, we download the reconciled EnsemblCompara gene trees, and extract the orthology information for the genomes being compared. Each group of genes of the concerned genomes that descended from the lowest common ancestor of the concerned genomes defines an ortholog group.

Some other tools and databases are also available for ortholog assignment among multiple genomes, such as the OrthoFocus program [32] and the PhylomeDB ortholog database [33]. However, OrthoFocus is a program to identify orthologs in family-focused studies and it is inapproriate for genome-scale comparisons. PhylomeDB is a major source for phylogeny-based orthology and paralogy prediction, covering about 5 million proteins in 717 fully-sequenced genomes. However, since it involves a large number of genomes in the comparison, we are unable to retrieve reconciled gene trees concerning only genes from genomes of interest to us. Instead, we are only provided with orthology relationship with respect to a “seed” genome. This means that we would need to use a single-linkage method to combine ortholog groups via “seed” genomes, which is clearly undesirable. Besides, PhylomeDB generally presents orthology as a many-to-many relationship. Without reconciled trees, it is hard for us to refine the relationship into a one-to-one relationship, which makes the comparison with our results very difficult. Moreover, PhylomeDB uses a data source different from Ensembl, and the conversion of gene names between the two databases could be quite non-trivial.

#### Results on Human, Mouse and Rat

Since human, mouse and rat are the best annotated genomes, we can use gene symbols to validate the ortholog groups assigned among the three genomes by different programs. The same validation method has been used in many other papers [5], [19], [20]. Note that since some gene symbols were assigned using information from certain orthology databases, we should take the validation results based on gene symbols with a grain of salt. By using gene symbols, we can define true ortholog groups (TPs), false ortholog groups (FPs), and unknown ortholog groups as follows. If an ortholog group contains genes that have different gene symbols, then this group is counted as an FP. If at most one of the genes in the group have gene symbols, then this group is counted as an unknown. Otherwise, we treat the group as a TP. An ortholog group is defined as *assignable* if its genes appear in at least two genomes and have exactly the same gene symbol. We use the same measurements *sensitivity* and *specificity* as defined in the simulation to measure the prediction accuracies of the three programs. The performance of the programs is shown in Table 1.

**Table 1:** Performance of the four programs on human, mouse and rat.

Program	Assignable TPs	TPs	FPs	Unknowns	Total	Sensitivity	Specificity
MultiMSOAR 2.0	15,598	14,051	2,399	2,919	19,369	90.08%	85.42%
MultiParanoid	15,598	13,697	2,609	2,328	18,634	87.81%	84.00%
Ensembl	15,598	13,474	2,495	2,091	18,060	86.38%	84.38%
Roundup	14,616	10,094	2,424	6,790	19,308	69.06%	80.66%

The low sensitivity of Roundup in Table 1 may be caused by the mapping of gene IDs from Roundup to Ensembl since quite a few of the genes in Roundup were mapped to the unknowns in Ensembl. Nevertheless, we can see that MultiMSOAR 2.0 achieves the best sensitivity and specificity among all four programs. This is mainly because MultiParanoid only considers sequence similarity when assigning ortholog groups, while Ensembl ortholog groups tend to include lots of lineage-specific duplicated inparalogs. Though Roundup is based on the reciprocal smallest distance algorithm, which is different from the reciprocal BLAST hits used in MultiParanoid, it fails to consider other information as well. In contrast, MultiMSOAR 2.0 combines gene order with sequence similarity, as well as phylogenetic information, and thus is able to make more accurate predictions.

#### Results on All Seven Mammalian Genomes

When comparing the seven mammalian genomes including human, chimpanzee, macaque, mouse, rat, cow, and opossum, we cannot validate the ortholog groups predicted by the three programs using gene symbols since not all of the genomes have been annotated with gene symbols. So, we only consider the common and different ortholog groups constructed by MultiMSOAR 2.0, MultiParanoid, Roundup and the Ensembl ortholog database. The comparison results are shown in Table 2 (since we are not able to find a good mapping from the data used in Roundup repository to the data used in Ensembl concerning all seven genomes, the comparison results with Roundup are not included in the table).

**Table 2:** Ortholog groups shared by MultiMSOAR 2.0, MultiParanoid and Ensembl on the seven mammalian genomes.

Programs	7 genomes	6 genomes	5 genomes	4 genomes	3 genomes	2 genomes
MultiMSOAR 2.0	12,034	3,772	1,337	584	875	3,195
MultiParanoid	11,397	3,311	1,127	609	800	2,728
Ensembl	13,566	2,002	493	270	363	991
MultiMSOAR 2.0 and MultiParanoid	9,075	2,237	633	239	348	1,483
MultiMSOAR 2.0 and Ensembl	8,722	1,003	225	104	131	524
MultiParanoid and Ensembl	8,438	983	237	117	143	587
All three programs	7,763	872	202	92	119	505

Table 2 shows the numbers of ortholog groups involving 2 to 7 genomes that were identified by MultiMSOAR 2.0, MultiParanoid and Ensembl. From Table 2, we can see that the numbers of ortholog groups found by all three programs are similar to each other for each number of genomes involved. Most of the ortholog groups identified by each of the three programs all involve seven genomes. Among such large ortholog groups identified by each program, more than a half (7,763) are shared by all three programs, which provides an indirect support for the ortholog groups found by MultiMSOAR 2.0. The large number of ortholog groups involving all seven genomes found by the three programs also manifests the evolutionary closeness of the seven mammalian species. The number of ortholog groups involving 4 genomes found by the three programs is pretty small here, since there is no subtree in the species tree consisting of exactly four species. Hence, an ortholog group of size four would have to involve gene losses. Since there is only one subtree consisting of three species (*i.e.*, human, chimpanzee, and macaque), most of the 875 ortholog groups of size 3 found by MultiMSOAR 2.0 (679, or about 77.6%) consist of genes from the three species. Similarly, 1,772/3,195 (55.46%) and 1,083/3,195 (32.49%) of the ortholog groups of size two consist of genes from mouse-rat and human-chimpanzee respectively, both of which are the closest pairs in the species tree.

### Conclusion

In this paper, we have extended the pairwise ortholog assignment system MSOAR 2.0 to a multi-genome ortholog assignment system MultiMSOAR 2.0. By comparing with the well known multi-genome ortholog assignment tool MultiParanoid on simulated data, we demonstrated that MultiMSOAR 2.0 achieves a significantly higher prediction accuracy. Our real data experiments on closely related mammalian genomes also show the superior performance of MultiMSOAR 2.0 over MultiParanoid, the multi-genome ortholog repository Roundup and the Ensembl ortholog database. Moreover, not only can MultiMSOAR 2.0 identify ortholog groups accurately, it can also provide accurate information about gene births, losses and duplications, which may shed additional insight on genome evolution.

## Supporting Information

Materials S1 Additional Experimental Results on Simulated Data.
(PDF)
