# Supplementary material for: MultiMSOAR 2.0: An Accurate Tool to Identify Ortholog Groups among Multiple Genomes
Source: PLoS One. 2011 Jun 21;6(6):e20892. doi: 10.1371/journal.pone.0020892 (PMC3119667; doi:10.1371/journal.pone.0020892)
Supplement: Materials S1 — Additional Experimental Results on Simulated Data. (PDF) [file pone.0020892.s001.pdf]

Materials S1

Additional Experimental Results on Simulated Data

The accuracies of MultiMSOAR 2.0 and Notung in inferring gene births, losses and duplications on simulated data are summarized in the following tables. In each table, the first number in a cell gives the accuracy of MultiMSOAR 2.0 while the second corresponds to Notung.

Table S1: Prediction accuracy when the parameter S (the number of species) is varied.

S	3	5	7	9	11
GeneBirth Sensitivity	95.00% / -	100.0% / -	100.0% / -	96.25% / -	87.00% / -
GeneBirth Specificity	71.00% / -	84.89% / -	84.64% / -	85.23% / -	93.90% / -
GeneDuplication Sensitivity	89.38% / 71.88%	89.06% / 61.56%	89.38% / 58.13%	87.66% / 55.47%	79.88% / 54.88%
GeneDuplication Specificity	99.33% / 59.61%	92.81% / 39.17%	92.70% / 22.65%	93.83% / 18.88%	94.64% / 17.10%
GeneLoss Sensitivity	47.50% / 0.00%	63.21% / 0.00%	66.67% / 0.83%	73.13% / 1.38%	84.63% / 2.00%
GeneLoss Specificity	93.33% / 0.00%	81.75% / 0.00%	77.63% / 0.48%	74.74% / 0.05%	52.74% / 0.11%

Table S2: Prediction accuracy when the parameter E (the number of evolutionary events) is varied.

E	5	10	15	20
GeneBirth Sensitivity	96.67% / -	100.0% / -	96.25% / -	96.88% / -
GeneBirth Specificity	76.38% / -	84.89% / -	76.78% / -	76.48% / -
GeneDuplication Sensitivity	91.88% / 60.00%	89.06% / 61.56%	85.83% / 62.08%	83.91% / 60.16%
GeneDuplication Specificity	96.91% / 37.61%	92.81% / 39.17%	95.17% / 32.69%	92.87% / 29.46%
GeneLoss Sensitivity	53.81% / 0.00%	63.21% / 0.00%	62.08% / 2.50%	45.42% / 2.59%
GeneLoss Specificity	74.17% / 0.00%	81.75% / 0.00%	69.92% / 0.13%	74.58% / 0.14%

Table S3: Prediction accuracy when the parameter μ (evolver branch length) is varied.

μ	0.01	0.03	0.05	0.07	0.15
GeneBirth Sensitivity	97.50% / -	97.50% / -	100.0% / -	98.75% / -	98.75% / -
GeneBirth Specificity	82.22% / -	79.75% / -	84.89% / -	78.44% / -	54.02% / -
GeneDuplication Sensitivity	86.56% / 61.88%	92.81% / 61.25%	89.06% / 61.56%	86.41% / 61.41%	95.00% / 58.75%
GeneDuplication Specificity	90.88% / 36.71%	93.80% / 37.96%	92.81% / 39.17%	92.94% / 37.48%	89.92% / 31.10%
GeneLoss Sensitivity	60.89% / 1.25%	65.00% / 0.00%	63.21% / 0.00%	59.55% / 0.00%	58.75% / 0.00%
GeneLoss Specificity	75.51% / 0.18%	89.11% / 0.00%	81.75% / 0.00%	82.79% / 0.00%	87.03% / 0.00%

Table S4: Prediction accuracy when the parameter α (the ratio of duplication events) is varied.

α	20%	30%	40%	50%	60%
GeneBirth Sensitivity	98.75% / -	97.50% / -	100.0% / -	100.0% / -	97.50% / -
GeneBirth Specificity	79.00% / -	81.50% / -	84.89% / -	80.00% / -	80.44% / -
GeneDuplication Sensitivity	93.13% / 61.25%	93.33% / 61.67%	89.06% / 61.56%	86.00% / 61.25%	87.29% / 61.25%
GeneDuplication Specificity	94.44% / 37.80%	95.32% / 36.75%	92.81% / 39.17%	94.44% / 37.49%	95.31% / 38.21%
GeneLoss Sensitivity	71.25% / 0.00%	67.50% / 0.00%	63.21% / 0.00%	64.58% / 0.00%	60.71% / 1.25%
GeneLoss Specificity	96.90% / 0.00%	90.00% / 0.00%	81.75% / 0.00%	75.32% / 0.00%	78.18% / 0.56%

A Graphical Illustration of Genome-Level Evolutionary Events

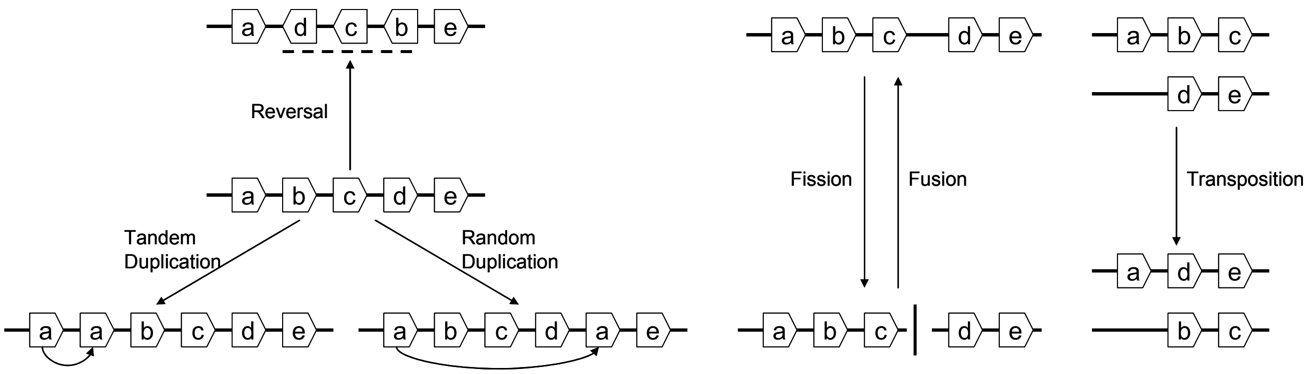

Figure S2: Genome-level evolutionary events considered in the simulation.
